# Neuro-hormonal effects of physical activity in the elderly

**DOI:** 10.3389/fphys.2013.00378

**Published:** 2013-12-20

**Authors:** Grazia D. Femminella, Claudio de Lucia, Paola Iacotucci, Roberto Formisano, Laura Petraglia, Elena Allocca, Enza Ratto, Loreta D'Amico, Carlo Rengo, Gennaro Pagano, Domenico Bonaduce, Giuseppe Rengo, Nicola Ferrara

**Affiliations:** ^1^Department of Translational Medical Sciences, University of Naples Federico IINaples, Italy; ^2^Division of Cardiology, Salvatore Maugeri Foundation, IRCCS, Scientific Institute of Telese Terme (BN)Telese Terme, Italy

**Keywords:** physical activity, elderly, sympathetic nervous system, renin-angiotensin-aldosterone system, brain natriuretic peptide

## Abstract

Thanks to diagnostic and therapeutic advances, the elderly population is continuously increasing in the western countries. Accordingly, the prevalence of most chronic age-related diseases will increase considerably in the next decades, thus it will be necessary to implement effective preventive measures to face this epidemiological challenge. Among those, physical activity exerts a crucial role, since it has been proven to reduce the risk of cardiovascular diseases, diabetes, obesity, cognitive impairment and cancer. The favorable effects of exercise on cardiovascular homeostasis can be at least in part ascribed to the modulation of the neuro-hormonal systems implicated in cardiovascular pathophysiology. In the elderly, exercise has been shown to affect catecholamine secretion and biosynthesis, to positively modulate the renin-angiotensin-aldosterone system and to reduce the levels of plasma brain natriuretic peptides. Moreover, drugs modulating the neuro-hormonal systems may favorably affect physical capacity in the elderly. Thus, efforts should be made to actually make physical activity become part of the therapeutic tools in the elderly.

## Introduction

Aging is a physiological process influenced by both genetic and environmental factors and the mechanisms involved mainly remains unknown. In the US, more than 3 million citizens are presently 85 years or older and the elderly population is probably expected to further rise by 2040. This escalation is due to increase in average age (thanks to improvements in diagnostic and therapeutic tools). Furthermore, the prevalence of most chronic age-related diseases will begin to increase considerably within the next two decades (Vogel et al., 2009). Consequently, it will be necessary to implement effective preventive measures against the progression of chronic diseases, focusing above all on life style measures. While no intervention has been shown to increase overall longevity, some seems to influence the aging process. Among these, physical activity exerts a crucial role, in addition to healthy balanced diet and psychosocial wellbeing (Gremeaux et al., 2012).

It is known that physical exercise beneficially affects the human body in a multifactorial way. Indeed, with the exclusion of diet modification, no other single intervention has a greater promise than physical exercise to reduce the risk of the most common chronic diseases at the same time. Regular physical activity has clearly been shown to reduce the risk of cardiovascular disease, stroke, hypertension, type 2 diabetes, obesity, osteoporosis, cancer (e.g., colon and breast cancer) and psychiatric pathologies as anxiety and depression. There is some evidence that exercise training also prevents or delays cognitive impairment and improves quality sleep, combating insomnia. Clinical guidelines also identify a role for exercise in the comprehensive management of dementia, chronic pain, congestive heart failure, stroke, prophylaxis of venous thromboembolism (Booth et al., 2000, 2011; Marciano et al., 2012; Paolillo et al., 2013).

Moreover, it is widely recognized that exercise also has important effects on diverse neuro-hormonal systems that can be altered in the elderly especially during the course of most common geriatric diseases. In this mini-review, we will explore how some of the most important systems regulating cardiovascular homeostasis can be affected by aging and how physical activity can have a crucial role in preventing this deterioration.

## Sympathetic nervous system in the elderly and its modulation by exercise training

The sympathetic nervous system (SNS) is a pivotal modulator of some important functions and particularly of cardiovascular and metabolic ones. Through the SNS, the central nervous system maintains the homeostasis after acute and chronic stimuli, as well as in response to the development of pathological conditions (Davy et al., 1995; Esler et al., 1995; Zincarelli et al., 2010). Many studies evaluated the effects of aging on SNS under basal conditions and in response to acute physical and mental-emotional stress. It has been established that SNS activity, as estimated from total plasma norepinephrine spillover, is greater in older healthy adults compared to young controls. This sympathetic overdrive in advanced age is related to the elevation in basal tonic activity rather than in response to stress and is mainly due to SNS outflow in peripheral tissues (Seals and Esler, 2000).

Some authors have hypothesized that one of the key mechanisms underlying this increase in tonic SNS activity in elderly is the increasing in total and abdominal adiposity and in circulating adipose-sensitive signals (e.g., leptin) and augmented brain noradrenergic activity (Femminella et al., 2013), as evaluated by measurements of norepinephrine spillover from the cerebrovascular circulation. It has been speculated that increasing in tonic SNS activity with aging could be an adaptive response to increasing accumulation of visceral body fat (Seals and Dinenno, 2004).

SNS over-activation has detrimental effects on the cardiovascular system mainly during clinical diseases such as congestive heart failure and essential hypertension. It has been demonstrated that the raise in SNS activity with aging contributes to increasing in arterial blood pressure observed in older adults. In addition, aging is related with impaired limb vasoconstrictor responsiveness to chronic SNS stimulation via α1-adrenergic receptors pathway (Dinenno et al., 1999). In particular, SNS overactivity and augmented local and systemic release of norepinephrine may stimulate the production of reactive oxygen species in the vascular wall by a α1-adrenergic-dependent mechanism, contributing to arterial wall hypertrophy. Moreover, increasing in SNS activity in the elderly also appears to have important effects on β-Adrenergic Receptor (β-AR) leading to some consequences on cardiovascular system. Particularly, chronic β-AR stimulation in aging affects vasodilatation, heart rate regulation and left ventricular contractility as demonstrated in both experimental animals and humans (Ferrara et al., 1997; Rengo et al., 2012c, 2013a). The mechanisms implicated in β-AR reduced responsiveness with aging seem involve both receptor and postreceptor elements of the β-adrenergic signaling. It has been suggested that the tonic raise in SNS activity with aging causes agonist-induced downregulation of β-ARs, desensitization of the β-ARs signaling pathway, and reduced vascular and cardiac tissue responsiveness to acute and chronic β-AR stimuli (Rengo et al., 2009b, 2012b,d; Lymperopoulos et al., 2012; Cannavo et al., 2013a; Salazar et al., 2013).

Regular physical activity reduces the risk of cardiovascular diseases, increases cardiovascular function in healthy subjects and in particular in patients with cardiovascular disease, is beneficial in controlling hypertension, can increase longevity in animal models and also quality of life in humans (Conti et al., 2012, 2013). Exercise exerts effect on cardiovascular risk factors mainly through reduction of platelet aggregation, increase in blood flow and oxygen delivery to skeletal muscle, improvement of cardio-respiratory fitness, reduction of body weight, improvement of lipid profile and increase in insulin sensitivity (Rinaldi et al., 2006; Rengo et al., 2010a, 2012a, 2013b,d; Barcelos-Ferreira et al., 2013).

Training-induced decreases in sympathetic activity may be beneficial in preventing arterial stiffening in hypertension; it has been recently demonstrated that in elderly hypertensives 12 weeks of training resulted in comparable reductions in blood pressure and improvements of endothelial function both in the presence and absence of beta-blockade, suggesting that drug therapy provided no additive benefit and did not affect the antihypertensive activity of exercise training (Fu et al., 2008).

However, the effects of exercise training on circulating catecholamines in elderly humans are controversial. In some studies, regular exercise does not seem to influence plasma norepinephrine levels in elderly subjects with normal plasmatic catecholamines, whereas exercise training decrease plasma norepinephrine concentrations in elderly humans with elevated baseline levels. In addition, exercise training reduces both systolic and diastolic blood pressure in young and elderly hypertensive subjects (Kohrt et al., 1993).

Physical activity could act in diminishing circulating catecholamines by a decrease in catecholamine biosynthesis. Tumer et al. demonstrated that long-term (10 weeks) treadmill exercise determinates adaptive changes in young rats that lead to a decrease in tyrosine hydroxylase expression and activity in the adrenal medulla, but the same training schedule failed to decrease the previously elevated tyrosine hydroxylase level and activity in the old rats (Tumer et al., 2001).

However, some authors hypothesize that forced modes of exercise, like treadmill training, may induce by themselves a stress response, and these stressor effects of forced exercise might be more evident in senescent animals, abolishing the beneficial effects of exercise training (Moraska et al., 2000). More recently, Erdos et al. have showed that age-related changes in the levels of catecholamine biosynthetic enzymes in the adrenal medulla and hypothalamus can be abolished with only 8% restriction in daily caloric intake, while life-long voluntary exercise does not have any additional effect over caloric restriction (Erdos et al., 2007; Lymperopoulos et al., 2008, 2009, 2011).

Our group has shown that in a chronic diseases characterized by SNS hyperactivity, such as heart failure, exercise training (Rengo et al., 2007, 2010a; Lymperopoulos et al., 2010; Cannavo et al., 2013b) has beneficial effects on curbing the cardiotoxic effects of sympathetic overactivation, exerting a similar (and/or complementary) neurohormonal action to that of β-blockers in combating the autonomic derangements that confound and aggravate chronic heart failure (Rengo et al., 2004, 2009a; Lymperopoulos et al., 2013). Thus, it could be hypothesized that regular physical activity can have beneficial effects also in the aging process through the modulation of the sympathetic tone (Leosco et al., 2007; Rengo et al., 2010b).

## Renin-angiotensin-aldosterone system in the elderly and its modulation by exercise

The renin-angiotensin-aldosterone system (RAAS) regulates cardiovascular functions under both normal and pathologic conditions, through receptors widely distributed in the whole body. RAAS increased activity is associated with the establishment and development of hypertension, cardiovascular events, and chronic kidney disease and drugs modulating this system are very important and diffused tools for the treatment of essential hypertension and other cardiac and vascular diseases. In the RAAS cascade angiotensinogen is cleaved by renin to form angiotensin I (AngI), which, in turn, is converted to angiotensin II (AngII) by angiotensin-converting enzyme (ACE) (Fyhrquist and Saijonmaa, 2008). AngII has direct action on renal tubular sodium retention and stimulate the secretion of aldosterone in the adrenal glands, which in turn determinates salt and water reuptake by the kidneys. Thus, the RAAS is a key system in blood pressure regulation through the modulation of body fluids and electrolyte concentrations. Moreover, the importance of AngII was mostly clear when some authors showed that deletions of the AT1A receptors or ACE genes significantly decrease systolic blood pressure in mice (Cole et al., 2000).

It has been shown in recent times that pharmacological inhibitors of the RAAS are also able to increase levels of the heptapeptide angiotensin-(1–7). This peptide is formed by cleavage of either AngI by various endopeptidases or from AngII by ACE and its actions counteracts the deleterious cardiovascular effects of AngII, as angiotensin-(1–7) induces vasodilation and facilitation of baroreflex sensitivity. These findings are particularly interesting because an imbalance in AngII and angiotensin- (1–7) levels is involved in aging and in several animal models of hypertension, a typical disease of the elderly (Iyer et al., 1998).

In addition to that, chronic therapies with ACE inhibitors or AT1 receptor blockers (ARBs) improves or prevents deficits in cardiovascular, metabolic and renal function during aging in normotensive and hypertensive rodents. A recent study has demonstrated that global disruption of the AT1A receptor in mice augmented survival through attenuation of age-related mitochondrial dysfunction and oxidative stress as well as upregulation of antiapoptotic genes (Benigni et al., 2009; Corbi et al., 2012).

During aging, AT1A receptor knockout mice also exhibit a lean phenotype and preserved bone mass and cerebral endothelial function (Arnold et al., 2013). These findings indicate that both ACE and the AT1 receptor might have detrimental consequences in age-related pathologies; however, the mechanisms involved in the beneficial effects of RAAS blockade or genetic deletion during aging remain unclear. Particularly, it is not completely clear whether these effects are peripheral or central: chronic therapies involving RAAS or global deletion of RAAS receptors and enzymes might not only influence brain regions involved in cardiovascular and metabolic control, but also block local AngII effects on peripheral tissues. Moreover, ACE inhibitors and ARBs change the balance of the RAAS augmenting angiotensin-(1–7) levels, and this may be important during aging (and consequently for old patients with heart disease and hypertension), when deficiency of this peptide also contributes to impairments in baroreflex sensitivity (Iyer et al., 1998).

Some authors have also shown that plasma renin activity and renin responsiveness decrease with age, possibly due to the effect of age-associated nephrosclerosis. Plasma aldosterone is also decreased during aging, leading to an augmented risk for hyperkalemia in older individuals, especially in old patients with decline in glomerular filtration rate (Turgut et al., 2010).

Pharmacological therapy with ACE inhibitors could improve physical function in elderly people. ACE inhibitors ameliorate endothelial function and may enhance muscle function by increasing muscle blood flow and glucose delivery. In particular, an observational study on elderly women with hypertension confirmed that ACE inhibitors slowed the decline in physical function and muscle strength when compared with other antihypertensive agents and in a randomized placebo-controlled trial, ACE inhibitors permitted a significant enhancement in exercise capacity among elderly patients with heart failure (Onder et al., 2002; Henriksen and Jacob, 2003). Although this effect may have been the result of improved cardiac function, ACE inhibitors may have direct effects on skeletal muscle. Furthermore, a recent study has also verified that the ACE inhibitor perindopril enhanced exercise functional capacity in elderly people without heart failure and maintained health-related quality of life (Sumukadas et al., 2007).

Some studies have also evaluated the possible correlations between ACE genotype, exercise training and blood pressure control. ACE plasma levels may be related to the insertion/deletion (I/D) polymorphism of the *ACE* gene, located on chromosome 17. Dengel et al. showed no significant interaction between genotype and aerobic training in relation to blood pressure, while another report showed that 10 weeks of aerobic exercise considerably decreased blood pressure only in patients with genotypes II and ID, and not in the subjects with the DD genotype of ACE (Dengel et al., 2002). Blanchard et al. showed a greater diastolic pressure fall in subjects with DD genotype compared to genotypes II and ID after mild aerobic training and Kim observed that, in adult women, the DD genotype showed greater diminution of diastolic pressure, compared to the II group, when performing aerobic and resistance exercise (Blanchard et al., 2006; Kim, 2009).

Limited data are available on the effects of exercise training on hormones plasma levels in healthy elderly. A previous study by Carroll et al. evaluated the effects of 6 months of endurance training on plasma hormones in the elderly. They showed that levels of adrenocorticotropic hormone, vasopressin, aldosterone, norepinephrine and epinephrine remained unchanged after the training (Carroll et al., 1995).

Emerging data demonstrate a pivotal role for the RAAS in modulation of autonomic nervous system pathways participating in the regulation of cardiovascular and metabolic functions, and this could be most relevant during aging, especially due to its role in blood pressure regulation.

## Brain natriuretic peptide (BNP) levels in elderly patients and the effects of exercise training

BNP is a 32 amino acid peptide and usually it is measured by its N-terminal fragment (NT-pro-BNP), since the N-terminal part seems to be more stable than BNP itself. BNP is a cardiac neuro-hormone secreted from the ventricles in response to volume expansion and pressure overload (myocyte stress) and has different systemic effects, such as vasodilation, augment in urinary volume and sodium output, and inhibition of SNS and RAAS systems. Increasing in plasma levels of BNP and NT-pro-BNP are generally related to a regional or global impairment of left ventricular (LV) systolic or diastolic function (LV wall stress). It has been demonstrated that BNP measurement is a reproducible and sensitive method to monitor LV systolic and diastolic dysfunction, and both BNP and NT-pro-BNP are strong predictors of morbidity and mortality in patients with heart failure and coronary heart disease (Hall, 2004; Kragelund et al., 2005). Moreover, plasma levels of BNP tend to be higher in older patients with or without cardiac dysfunction and the increase in elderly could be explained by the loss of clearance receptors with aging or by the recent evidence that BNP and NT-proBNP correlate with renal function and decreased glomerular filtration rate can markedly has a significant prevalence in the elderly population (Krupicka et al., 2009).

In addition to that, BNP increases with age and has a prognostic value in healthy elderly subjects, in functionally impaired patients and in elderly patients with cardiovascular diseases. Augmented BNP levels have detrimental systemic effects on skeletal muscle (reduced blood flow, cachexia), myocardium (adverse LV remodeling, decreased cardiac contractility) and vasculature (development of atherosclerosis, oxidative stress, endothelial cell apoptosis and adverse vascular remodeling) (Wallen et al., 1997; Anker and von Haehling, 2004).

Some authors have showed that physical activity is able to reduce BNP levels in heart failure patients, and these results have been also confirmed in recent systematic reviews and meta-analysis (Smart and Steele, 2010; Smart et al., 2012; Rengo et al., 2013c; Savarese et al., 2013). Data on elderly heart failure patients also demonstrate that exercise training is associated with a decrease of NTpro-BNP plasma levels and with an improvement of cardiovascular function, with ameliorated LV systolic function and intraventricular pressure (Giallauria et al., 2006). A recent study has evaluated the relationship between objectively measured daily walking duration in the elderly and cardiovascular biomarkers of inflammation, cardiac dysfunction and renal impairment, including NT-proBNP. In this work physical activity has shown beneficial effects on cardiovascular risk factors like blood pressure, blood lipids and inflammation markers, which were inversely associated with walking (Klenk et al., 2013). Moreover, objectively measured walking duration showed an inverse dose-response relationship with the circulating biomarkers of cardiac dysfunction.

## Conclusions

Though aging is inescapable, yet its effects can be counteracted. It is well-established that sedentary lifestyles accelerate aging by increasing risks of chronic diseases and reducing the average life expectancy. Exercise can help to ameliorate the quality of life, mainly preserving functional reserve in the elderly. Numerous studies have shown that maintaining regular and minimal exercise training decreases the risk of cardiovascular morbidity and mortality and ameliorates the modulation of neuro-hormonal systems regulating cardiovascular pathophysiology.

Thus, physical inactivity remains a major health challenge for the elderly and, although the benefits seem to be linked to the intensity of training, exercise prescription still needs to be clarified to enable the scientific community to develop precise recommendations.

### Conflict of interest statement

The authors declare that the research was conducted in the absence of any commercial or financial relationships that could be construed as a potential conflict of interest.
